# The complete chloroplast genome sequence of *Castanopsis fordii* Hance (Fagaceae)

**DOI:** 10.1080/23802359.2023.2167477

**Published:** 2023-02-10

**Authors:** Ya-Feng Wang, Yong-Lin Huang, Rui-Jie He, Bing-Yuan Yang, Zhang-Bin Liu

**Affiliations:** a Guangxi Zhuang Autonomous Region and Chinese Academy of Sciences, Guangxi Key Laboratory of Plant Functional Phytochemicals and Sustainable Utilization, Guangxi Institute of Botany, Guilin, China

**Keywords:** *Castanopsis fordii*, chloroplast genome, Fagaceae, phylogenetic analysis

## Abstract

*Castanopsis fordii* Hance 1884 is a typical evergreen broad-leaved forest plant in the south subtropical and middle subtropical regions of China. It has high utilization value in wood production and soil erosion protection. Here, we first reported and characterized the complete chloroplast (cp) genome sequence of *C. fordii* based on Illumina paired-end sequencing data. The complete cp genome sequence of *C. fordii* was 160,853 base pairs (bp) in length which contained two inverted repeats (IRs) of 25,699 bp separated by a large single-copy (LSC) and a small single copy (SSC) of 90,474 bp and 18,981 bp, respectively. The cpDNA contained 129 genes, comprising 85 protein-coding genes, 36 tRNA genes, 8 rRNA genes. The overall GC content of the plastome was 36.8%. Phylogenetic analysis base on 14 chloroplast genomes indicated that *C. fordii* was closely related to the species *C. tibetana* and *C. concinna* in Fagaceae.

## Introduction

*Castanopsis fordii* belongs to the genus *Castanopsis* of Fagaceae family is naturally distributed in southeastern China with the Nanling Mountains as the distribution center, and is often the main tree species constituting the local evergreen broad-leaved forest (Lian Bei et al. 2018). *C. fordii* with straight trunk, fast growth and reddish-brown heartwood is a common wood species in southern China and has high economic value (Xu et al. 2010). In this study, we reported the complete chloroplast (cp) genome of *C. fordii* based on Illumina pair-end sequencing data, which was helpful for well understanding its characteristics and the origin in evolution.

## Materials and methods

**Figure 1.**
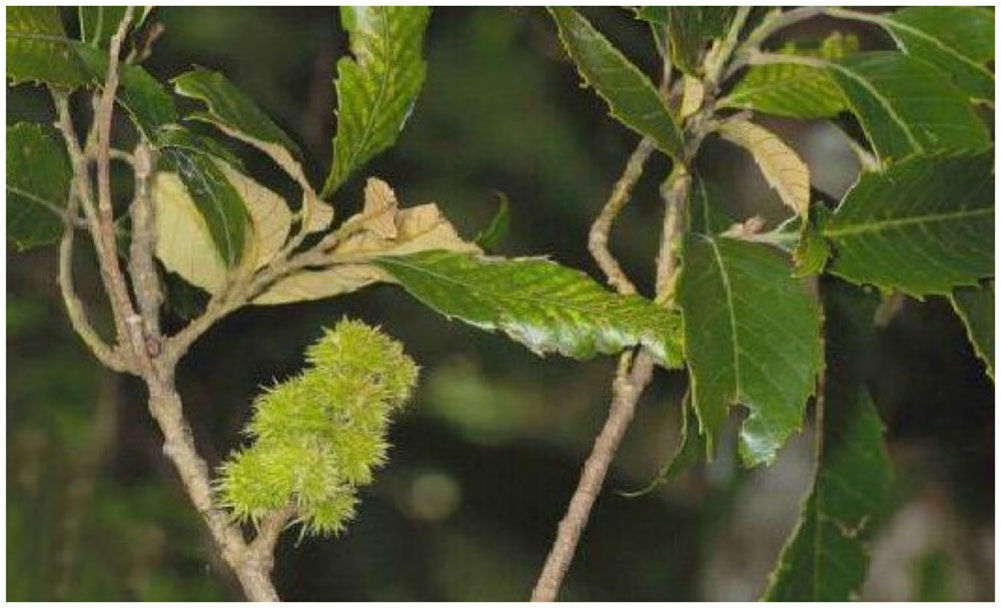
The Sample picture of *C. fordii.* The picture taken by Huang Yonglin in the Haiyang Mountain Nature Reserve.

Fresh leaves of *C. fordii* (Figure 1, Yong-lin Huang, hyl@gxib.cn) were collected from the Haiyang Mountain nature reserve in yangshuo of Guangxi province, China (24°59′ N, 110°55′ E). The voucher specimen has been deposited in the Guangxi Key Laboratory of Plant Functional Phytochemicals and Sustainable Utilization, Guangxi Institute of Botany (http://www.gxib.cn/, Yong-lin Huang, hyl@gxib.cn) under the voucher number MZ 20211223. DNA extraction from fresh leaf tissue was randomly disrupted into 300–500 bp by the Covaris ultrasonic breaker for library construction. The constructed library was sequenced using Illumina NovaSeq6000 platform, yielding approximately 2GB data. These trimmed reads were assembled by NOVOPlasty version 4.2 (Dierckxsens et al. 2016). The assembled genome was annotated using PGA (https://github.com/quxiaojian/PGA) (Qu et al. 2019) and Geseq with default settings (https://chlorobox.mpimp-golm.mpg.de/geseq.html) (Tillich et al. 2017). The annotation result was drawn with the online tool OGDRAW default parameters (https://chlorobox.mpimp-golm.mpg.de/OGDraw.html) (Greiner et al. 2019) (Figure 1).

## Results

**Figure 2.**
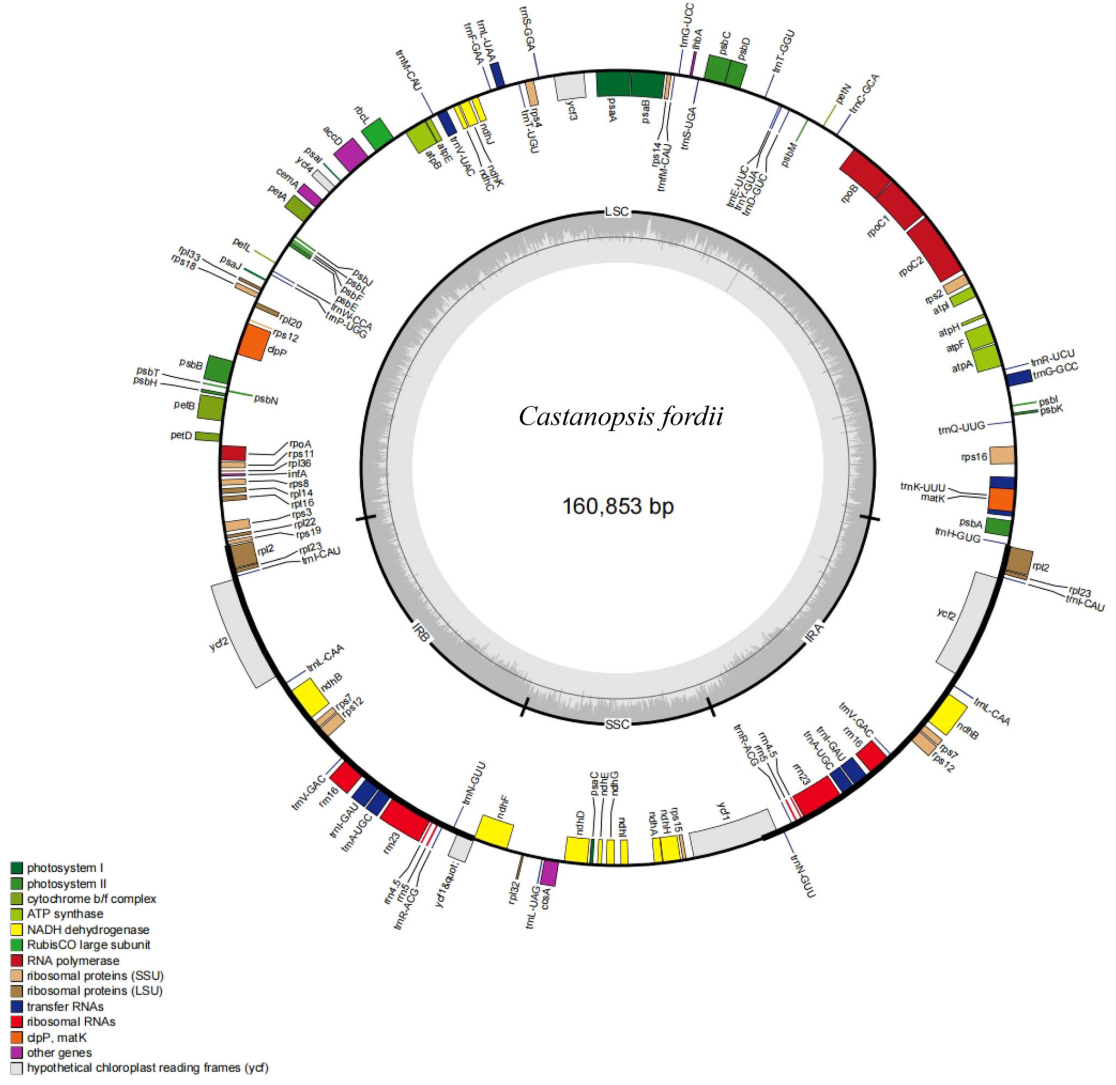
The Genome map of *C. fordii*. Genes inside the circle are transcribed clockwise, and those on the outside are transcribed counter-clockwise. Genes are colored according to functional categories. The darker grey area in the inner circle corresponds to GC content, whereas the lighter grey corresponds to AT content.

The complete cp genome sequence of *C. fordii* was 160,853 base pairs (bp) (Figure 2) in length which contained two inverted repeats (IRs) of 25,699 bp separated by a large single-copy (LSC) and a small single copy (SSC) of 90,474 bp and 18,981 bp, respectively. The cpDNA contained 129 genes, comprising 85 protein-coding genes, 36 tRNA genes, 8 rRNA genes. Most of the genes occurred in a single copy; however, six protein-coding genes (*ndh*B, *rpl*2, *rpl*23, *rps*12, *rps*7 *and ycf*2), seven tRNA genes (*trnl-*CAU, *trn*V-GAC, *trn*R-ACG, *trn*L-CAA, *trnl-*GAU, *trn*N-GUU and *trn*A-UGC), and four rRNA genes (4.5S, 5S, 16S and 23S) were totally duplicated. The over all GC content of the plastome was 36.8%, and the corresponding values in LSC was 35.07%. No gene has not been predicted. The annotated chloroplast genome of *C. fordii* has been deposited in GenBank with accession number ON710841.

**Figure 3.**
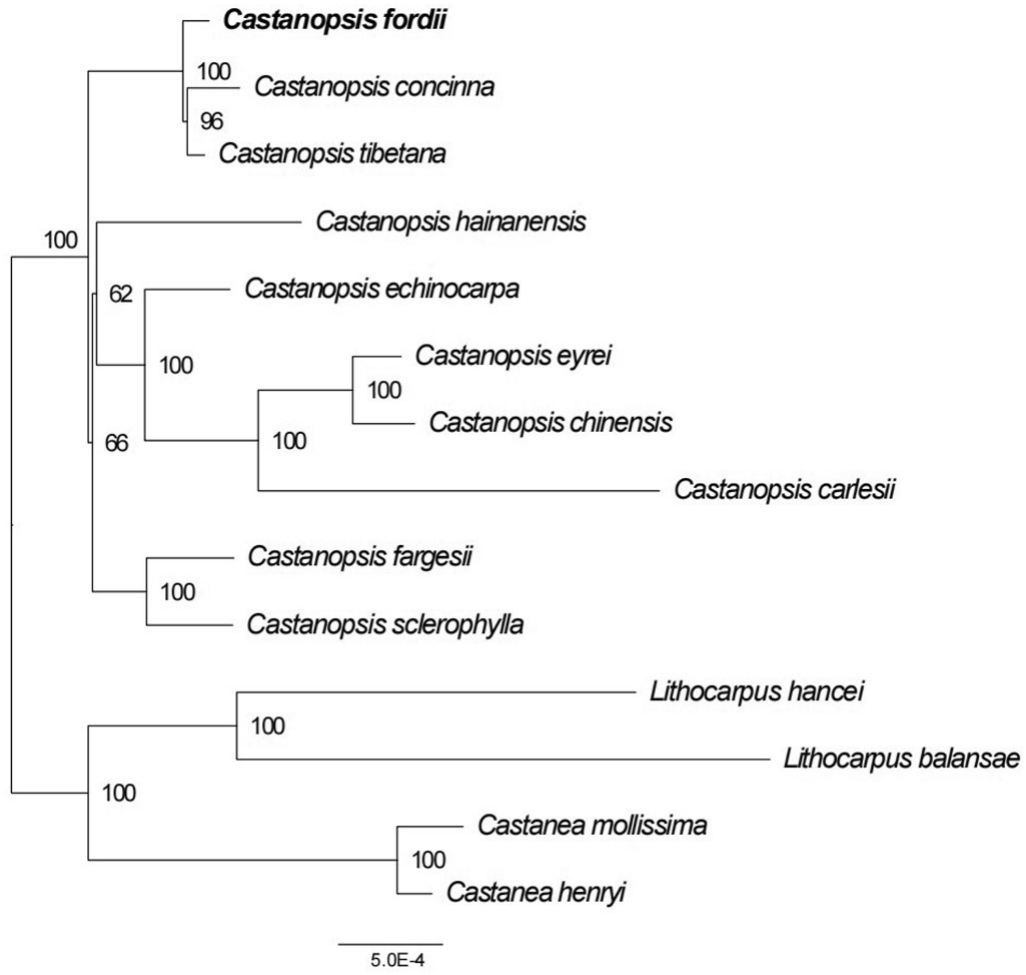
The ML phylogenetic tree based on fourteen complete chloroplast genomes. Maximum-likelihood phylogenetic tree was constructed by GTRCATI Model of RAxML based on complete cp genomes. The sequence type is the nucleotide sequence of the conservative coding gene. The bootstrap check value is 100. Numbers close to each node are bootstrap support values. Accession number: *Castanopsis concinna* KT793041.1 (Shi et al. 2022), *Castanopsis hainanensis* MG383644.1 (Ogoma et al. 2022), *Castanopsis fargesii* NC_047230.1 (Li et al. 2022), *Castanopsis carlesii* NC_057119.1 (Wang et al. 2022), *Castanopsis sclerophylla* MT627605.1 (Miao et al. 2020), *Castanopsis echinocarpa* NC_023801.1 (Guo et al. 2022), *Castanopsis tibetana* ON710842 (Sanchez-Puerta and Abbona 2014), *Castanopsis eyrei* ON550006, *Castanopsis chinensis* ON500675, *Castanopsis fordii* ON710841 (Li et al. 2022), *Lithocarpus balansae* KP299291.1 (Shi et al. 2022), *Castanea henryi* KX954615.1 (Shi et al. 2022), *Castanea mollissima* NC_014674 (Jansen et al. 2011), *Lithocarpus hancei* MW375417.1 (Shi et al. 2022). *Castanopsis eyrei* and *Castanopsis chinensis* have no publications and can only cite the sequences by accession numbers.

In order to reveal the phylogenetic position of *C. fordii* with other members of Fagaceae, a phylogenetic analysis was performed by MAFFT v7.158b (Katoh and Standley 2013) based on 11 complete cp genomes of Fagaceae, and *Lithocarpus hancei*, *Lithocarpus balansae* as outgroups. Then, the phylogenetic tree with 1000 ultrafast bootstrap (UFBoot) replicates (Minh et al. 2013) was constructed by RAxML. As shown in the ML phylogenetic tree (Figure 3), the genus *Castanea* and *Lithocarpus* formed a monophyletic clade with high bootstrap value, respectively. *C. fordii* was closely related to *C. concinna* and *C. tibetana*. These results based on complete cp genome reported here will lay a basis for the study of phylogeny, phylogeography and population genetic diversity of *C. fordii*.

## Discussion and conclusion

The chloroplast genome structures of the Fagaceae species that have been studied all contain one LSC region one SSC region and two IR regions, which is consistent with the basic structural characteristics of angiosperm chloroplast genomes. At the same time, it was found that the variation in the LSC region accounted for the vast majority of the variation in the whole genome, and the difference in the length of the whole genome was mainly caused by the difference in the length of the LSC. In this study, a phylogenetic tree was constructed based on the complete cp genomes sequences of 14 Fagaceae species. The classification of each species was basically consistent with the traditional taxonomy. This study lays the foundation for further studies on the evolution of Fagaceae genomes.

## Data Availability

The genome sequence data that support the findings of this study are openly available in GenBank of NCBI at [https://www.ncbi.nlm.nih.gov] (https://www.ncbi.nlm.nih.gov/) under the accession no. ON710841. The associated BioProject, SRA, and Bio-Sample numbers are PRJNA864116, SRR20726142, and SAMN30074217 respectively.”

## Sampling statement

Our materials are not listed in the Wild Plants Under State Protection in China, so we need not any permission or license to collect them; and we only sampled a few leaf materials for molecular experiment and didn’t damage any plant.
